# Address of the Chairman of the Section on Stomatology, American Medical Association

**Published:** 1903-06

**Authors:** M. L. Rhein

**Affiliations:** New York City


### ADDRESS OF THE CHAIRMAN OF THE SECTION ON STOMATOLOGY, AMERICAN MEDICAL ASSOCIATION.[1]

BY M. L. RHEIN, M.D., D.D.S., NEW YORK CITY.

In officially opening the meeting of the Section on Stomatology, I desire to tender to the members my high appreciation of the honor conferred in asking me to preside over your deliberations. It has been the aim of our Section to encourage the membership of men who are deeply interested in the scientific advancement of this specialty of medicine. On this account our numerical strength has not been great, and I trust that the members will be prompt in attendance at the opening of all the sessions, so that the magnificent programme prepared for us by our worthy secretary will receive the attention and discussion which it merits.

It is unnecessary for me to take up your time with a summary of the scientific work of the year. It speaks for itself in our professional literature with which you are familiar. Our daily occupation as stomatologists includes the treatment of diseases of the oral cavity, primarily with a view to the preservation in a state of health of the teeth, on account of the great value they bear to the health of the general body. The art and handicraft of dentistry has taught us how to cope with dental caries successfully, so that no person in the civilized world at the present day need lose a single tooth from this cause alone.

Many people, however willing they may be to employ our services, are constantly losing teeth in consequence of various patho-

---

[1] Abstract of Address delivered before the Section on Stomatology, American Medical Association, at New Orleans, May 5, 1903.

logical disturbances of the peridental tissues. The symposiums held in past years by this Section on interstitial gingivitis, pyorrhœa alveolaris, etc., indicate how well we recognize the increasing number of teeth lost by this means. When this lack of inherent vitality of the tooth's periosteum has reached a certain stage, where not only seven-eighths of the root surfaces are covered by nodular secretions, but retrograde metamorphosis in its peculiar way is working towards sepsis and the destruction of cell tissue, we realize that such affected teeth are beyond human intervention. We also know that these very teeth, doomed to be lost as they are when too little vital connection is left, could have been preserved indefinitely if proper hygienic care and treatment had been instituted at the right time and kept up in a systematic way at regular intervals.

Preventive medicine, with its accompanying benefits, has been well understood since the time that Jenner proclaimed the virtues of vaccination. Since then prophylaxis has invaded the domain of nearly every disease, and its science and practice is daily increasing and its field of usefulness becoming better known.

Its value in the province of stomatology is well known to the poorest dentist in the land. The cleansing and polishing of every portion of exposed tooth surface, at regular intervals, in such a way as to preserve the tone and health of the contiguous capillary circulation will indefinitely preserve the life and stability of the human teeth. With a full knowledge of the inestimable value resulting from such systematic treatment, it is painful to admit that, as a rule, the general public get absolutely no benefit from the knowledge of the profession on this subject.

Like many other virtues, prophylactic treatment of the teeth is well understood by all dentists and practised by only a few stomatologists. It is scarcely necessary for me to attempt to draw a pen-picture of what the dentist does under the pretence of cleaning teeth when requested to perform this service, or to speak of the wilful neglect thereof by the majority when no especial demand is made by the patient. You have all examined the mouths of such patients, who inform you that for years their mouths have had the constant care of Dr. X, and he has cleaned their teeth every year. You have found secretions of years deposited on the roots. The more posterior and inaccessible the teeth, the greater the extent of deposits. You are not surprised at finding such teeth becoming loose, with a surrounding pericementum more or less inflamed, and corresponding loss of soft tissue from superficial necrosis.

They come to us in all stages of tissue loss, from those easily cured to those irretrievably lost. You can not fail to recognize the picture, neither can you fail to place the responsibility for this wholesale destruction of human teeth upon the dentist, who has failed to apply the principles of preventive medicine.

Are we to assume from a study of this sad picture that our calling is made up of men lacking in character and principle? Most emphatically no; in no specialty will be found men with higher attributes of character, in every sense of the word, than in this of ours.

There are very good reasons why this most important duty to our patients is seriously neglected. The necessity for performing the operations immediately necessary for the repair of existing lesions in tooth-structure and adjacent tissue is of paramount importance. They are more than sufficient to take up all the time of the man with an average practice. The difficulty of receiving commensurate pay for the hours of time required in faithfully carrying out the treatment by prophylaxis brings up the question of expediency. It is true that Dr. Smith, of Philadelphia, claims to personally give his patients this treatment at regular intervals. If an effort were made to follow out this method in an average practice, there would be time left for nothing else. It certainly is the consensus of professional opinion, that the busy practitioner cannot give up his valuable time for this tedious, monotonous, and irksome labor, however important it may be for the salvation of the human teeth. A small number of us have tried to solve this important problem by employing an assistant practitioner to attend to this department. In the judgment of your chairman, who has tried this method for twelve years, it has failed to satisfactorily solve the problem.

The employment, in a private office, of a graduate to make a specialty of this work is very likely the best remedy we have had at our disposal up to the present time. The greatest objection to the plan is the inability to retain a graduate possessing ordinary ambition and talent a very great length of time in this position. It is impossible to obtain satisfactory results in this treatment if it is necessary every year or two to place a new assistant in this department. The patients object to these changes, and cannot fail to contrast the work of the new assistant, who has to be freshly taught the character of the work desired, with the final work of the last assistant, which has become well-nigh faultless from a couple of years of constant practice. In discussing this subject with prominent men it has been generally conceded that far better results could be obtained if suitable female assistants, not graduates, were especially trained and employed for this work.

In some of the States the examining boards have threatened prosecution for infraction of the dental law if such a course be pursued; consequently it has not been adopted, much to the loss of the general public.

This brings up the question whether the cleansing and polishing of the teeth, massaging the gums, and the application of remedial agents by non-graduate assistants, under specific prescriptions and directions of the attending stomatologists, are infractions of the law.

It might as well be said that the administration of drugs hypodermatically, the introduction of sounds and catheters, lavage and enemata, the dressing of wounds, and numerous other services performed under medical direction by trained nurses are all infractions of the medical law. Take away the trained nurses from the physician and surgeon and it would be apparent at a glance how it would interfere with the number of his cases, to say nothing of the inferiority of the resulting attention, which is such an important adjunct whenever the nurse is in attendance.

If this correctly describes existing conditions, it follows as a natural conclusion that the problem of how to systematically carry out the prescriptions of dental prophylaxis would be easily solved by the introduction of the trained dental nurse.

In the State of New York, during the present session of the Legislature, a law is being passed placing the trained nurse under the jurisdiction of the State Board of Regents, which necessitates that they should possess the proper qualifications in education and practice. In view of the high esteem held for the work of the trained nurse, it appears remarkable that the sphere of her usefulness has not long since been extended to our own specialty.

It would be an easy matter to add to the training-schools for nurses a department of dental nurses. Applicants for admission to such a course should be required to pass a satisfactory preliminary examination. Outside of the general didactic instruction which they would receive, they should obtain additional instruction in regard to the oral cavity, etc., from a stomatological member of the school's faculty. They would also receive their manual training under the same supervision, and in the hospital material they would find ample opportunity for perfecting their working technique.

It is difficult to estimate in advance how much benefit would accrue to a large percentage of hospital cases if their mouths could be properly cleansed and rendered sterile.

Would it be claiming too much to say that there are serious conditions where such treatment properly administered might prove the turning-point towards recovery, or in organic diseases towards a material improvement in the general condition? Perhaps their greatest sphere of usefulness would be to place the mouths of patients in a sterile condition, preliminary to undergoing surgical operations. When we consider all the devices used in the modern operating-room, all devoted to obtaining ideal aseptic conditions; when we consider the patient anæsthetized in an atmosphere barren of pus germs, with the single exception that too frequently the patient's mouth is redolent of putrescent filth, washed with secretions of pus; when we consider such conditions, with a celebrated surgeon about to enter into the digestive tract, can we longer question the value of the preliminary treatment by the dental nurse in such a case? Then there is the infirmary practice connected with all the large hospitals. Here is a field for the doing of the greatest good to this vast class of unfortunates. The cleansing of the mouths of properly selected patients in the dispensaries, combined with the proper education for preserving oral hygienic conditions, would be of greater value in the uplifting of the masses than any other means at present employed.

Having graduated from the training-school, it would be in keeping with our other laws to compel the nurses to pass a State board examination. The passing successfully of such an examination would then entitle them to be registered as trained dental nurses. Being so registered, they would be able to practise their profession in private life. By that is not meant the fact that they would be licensed to go around indiscriminately cleansing the mouths of the people.

Their license to practise dental nursing should mean that they are permitted to cleanse, polish, and medicate the dental territory only under the prescription of the patient's attending stomatologist.

As chairman of your Section, I ask your approval for this means of placing within reach of the human race the boon of prophylaxis in stomatology. There are three good reasons why it should receive your endorsement. First it will tend materially towards the public good. Secondly, it will open to womankind a new vocation second to none in desirability. Thirdly, it will materially aid the stomatologist in the quality of his results.

